# Modern Management of Clinical Chorioamnionitis

**DOI:** 10.1155/S1064744995000457

**Published:** 1995

**Authors:** Thomas Westover, Robert A. Knuppel

**Affiliations:** 1 Division of Maternal-Fetal Medicine Department of Obstetrics, Gynecology, and Reproductive Sciences Robert Wood Johnson Medical School/St. Peter's Medical Center University of Medicine and Dentistry of New Jersey New Brunswick NJ USA; 2 Department of Obstetrics and Gynecology Cooper Hospital Three Cooper Plaza, Suite 211 Camden NJ 08103 USA

## Abstract

Clinical chorioamnionitis continues to contribute to fetal and maternal morbidity and mortality. Significant advances have been made in the last 20 years in understanding the pathophysiologic processes leading to chorioamnionitis. This review addresses the history, incidence, pathophysiology, host defenses, risk factors, diagnosis, and maternal and neonatal management of clinically evident chorioamnionitis. After a detailed review of the physiologic processes leading to clinical chorioamnionitis and sepsis, we present a modern management scheme designed to optimize perinatal outcome for both mother and fetus.


Infectious Diseases in Obstetrics and Gynecology 3:123–132 (1995)
© 1995 Wiley-Liss, Inc.

### Modern Management of Clinical Chorioamnionitis

**Thomas Westover and Robert A. Knuppel**

*Division of Maternal-Fetal Medicine, Department of Obstetrics, Gynecology, and Reproductive Sciences, Robert Wood Johnson Medical School/St. Peter's Medical Center, University of Medicine and Dentistry of New Jersey, New Brunswick, NJ*

### Abstract

**Clinical chorioamnionitis continues to contribute to fetal and maternal morbidity and mortality. Significant advances have been made in the last 20 years in understanding the pathophysiologic processes leading to chorioamnionitis. This review addresses the history, incidence, pathophysiology, host defenses, risk factors, diagnosis, and maternal and neonatal management of clinically evident chorioamnionitis. After a detailed review of the physiologic processes leading to clinical chorioamnionitis and sepsis, we present a modern management scheme designed to optimize perinatal outcome for both mother and fetus.** © 1995 Wiley-Liss, Inc.

Key words

Fever, amniotic infection, placenta, pregnancy, intrauterine infection

Intrauterine infection, either antepartum or intrapartum, continues to contribute to perinatal and maternal morbidity and mortality. A number of terms have been used to describe this condition including clinical chorioamnionitis, intrapartum fever, amnionitis, amniotic-fluid infection, and intraamniotic infection. Unfortunately, these terms have been used by different investigators to characterize 3 separate clinical processes: 1) histologic chorioamnionitis in the absence of clinical symptoms; 2) positive amniotic-fluid culture assessed at the time of amniocentesis for preterm labor or premature rupture of the membranes; and 3) "clinically evident" infection manifested by fever with localizing signs on physical examination. This review will address the history, incidence, pathophysiology, host defenses, risk factors, diagnosis, and management of intrauterine infection with an emphasis on "clinically evident" infection.

Clinical chorioamnionitis is diagnosed in the presence of clinical symptomatology: fever >38°C accompanied by uterine tenderness; maternal or fetal tachycardia; and malodorous, purulent amniotic fluid. Labor may or may not be present and the membranes may or may not be ruptured. Any of these physical findings may be absent depending on the severity and duration of the infection, virulence and inoculum size of the organism, integrity of the host defense mechanisms, and presence of masking factors such as regional anesthesia. The diagnosis should be confirmed by histologic examination of the placenta. The clinical usefulness of obtaining intrapartum amniotic-fluid cultures or placental cultures after delivery remains unknown. In addition, the effect of intrapartum antibiotics on the success rate of amniotic-fluid or placental cultures remains unclear. However, if desired, the obstetrician may, in the delivery room, manually separate the amnion from the chorion and obtain a culture of the fetal surface of the chorionic plate to optimize the yield from placental cultures and minimize the isolation of contaminant vaginal flora.

The presence of histologic chorioamnionitis does not imply that clinical chorioamnionitis was present

Address correspondence/reprint requests to Dr. Thomas Westover, Department of Obstetrics and Gynecology, Cooper Hospital, Three Cooper Plaza, Suite 211, Camden, NJ 08103.

Received November 14, 1994

Accepted July 25, 1995

Review Article prior to delivery. Many authors have examined the frequency with which histologic chorioamnionitis is found in febrile and afebrile parturients. In one of the largest prospectively evaluated series to date, Mueller-Heubach et al.[1] examined over 1,800 consecutive singleton placentas in a blinded fashion. Standardized cuts of the chorionic plate were examined, but neither the membrane roll nor umbilical cord was examined. Histologic chorioamnionitis was present in 18% of the term deliveries vs. 32% of the preterm deliveries. Other series have noted the incidences of histologic chorioamnionitis to be 15%–80%, depending on the population and gestational age studied and whether the sections were taken from the chorionic plate alone or from the chorion, membrane roll, and umbilical cord. Grading classifications of the severity of the inflammatory infiltrate have also varied considerably. Therefore, no definitive conclusions can be made regarding the effect of placental histology on fetal or neonatal prognosis. However, 2 observations have been consistently noted in the literature: severe histologic chorioamnionitis is infrequently found in afebrile gravidas and, if funisitis is present, severe chorioamnionitis will be found in the chorionic plate and membrane roll.

### HISTORICAL NOTES

In 1962, Russell and Amadeus[2] reviewed 131 consecutive cases of chorioamnionitis at a large obstetric institution and noted a maternal death rate of 3.8% secondary to sepsis. Gibbs and Locke,[3] in a review of maternal deaths in Texas from 1969–1973, reported that 10 of 501 maternal deaths were secondary to chorioamnionitis. The Medical Society of New Jersey Subcommittee on Maternal Mortality[4] reviewed 115 maternal deaths for the years 1985–1989 and noted no maternal deaths secondary to chorioamnionitis and sepsis during a period in which approximately 500,000 live births were recorded. We have observed no maternal deaths in a series of over 200 cases of clinical chorioamnionitis managed at our tertiary-care institution from 1990–1994. It would appear that advances in the understanding and management of chorioamnionitis along with improvements in critical-care medicine have led to decreases in the case fatality ratio for chorioamnionitis.

### INCIDENCE

Obstetric texts have stated in the past that the incidence of clinical chorioamnionitis is approximately 1–2%. Retrospective series at large indigent institutions in the 1970s revealed a 0.5–0.8% incidence of clinical chorioamnionitis. Conversely, 2 prospective series in the mid-1980s at teaching hospitals noted incidences of 4.2 and 10.5%.[5,6] It is unclear if these reported differences reflect actual changes in the incidence of the disease as a result of evolving obstetric practices (such as frequent invasive fetal monitoring during the active management of labor) or if they reflect the vagaries of disease detection in retrospective vs. prospective evaluations. The incidence of intrapartum chorioamnionitis varies according to the presence or absence of a number of classically described risk factors such as socioeconomic status, parity, duration of ruptured membranes and labor, presence of internal fetal monitoring systems, gestational age at delivery, number of vaginal examinations, and abnormalities in labor progression (see Risk Factors below).

Infrequently, clinical chorioamnionitis results from an invasive procedure such as amniocentesis, chorionic-villus sampling, cordocentesis, or cerclage. The risk of clinical infection after amniocentesis or chorionic-villus sampling is quite low, and large series have noted incidences of approximately 1 in 1,000 procedures.[7] These infections typically lead to pregnancy loss at a pre-viable gestation. More extensive and time-consuming procedures such as cordocentesis and intravascular transfusion may be associated with infection rates of 1% to as high as 5%.[8] Elective prophylactic cerclage is generally considered to carry an infectious complication rate of 1–2%, while emergency cerclage placement in the face of advanced cervical dilation and bulging membranes may be associated with rates of clinical chorioamnionitis as high as 25%.[9,10]

### PATHOPHYSIOLOGY

Clinical chorioamnionitis typically results from the ascension of organisms from the vagina to the uterine cavity after rupture of the membranes. Occasionally, infection may occur because of hematogenous dissemination of bacteria through the bloodstream. For example, *Listeria monocytogenes* has been reported to result from a blood-borne infection after the ingestion of contaminated food.[11] Even more rarely, bacterial infection with organisms such as *Streptococcus pyogenes* (group A streptococcus), *Neisseria gonorrhoeae*, *Campylobacter*, and

**TABLE 1. Distribution of microbes in 408 cases of intraamniotic infection[a]**

| Microbe | No. | % |
|---|---|---|
| Group B streptococci | 60 | 15 |
| Enterococci | 22 | 5 |
| *Escherichia coli* | 33 | 8 |
| *Gardnerella vaginalis* | 99 | 24 |
| Other aerobic gram-negative rods | 21 | 5 |
| Peptostreptococci | 38 | 9 |
| *Bacteroides bivius* | 120 | 29 |
| *Fusobacterium* sp. | 23 | 6 |
| *Bacteroids fragilis* | 14 | 3 |
| *Mycoplasma hominis* | 125 | 31 |
| *Ureaplasma urealyticum* | 195 | 47 |

[a]Data from Gibbs et al.[15]

*Streptococcus pneumoniae* may occur in the absence of labor or ruptured membranes if they ascend to the amniotic cavity.[12–14] It is unclear if these types of infections reflect a subcategory of high-virulence organisms or an alteration in local or systemic host factors.

The bacterial composition of the amniotic fluid in clinical chorioamnionitis associated with ruptured membranes appears to be polymicrobial. In 1982, Gibbs et al.[15] reported the microbiologic constituents of the amniotic fluid of patients with clinical chorioamnionitis and matched controls. Seventy percent of the patients with clinically evident infection had >100 CFU/ml of virulent organisms compared with only 8% of the controls. The most common organisms noted were anaerobes and group B streptococci. Gravett et al.[16] have noted similar findings. The distribution of organisms in over 400 cases of chorioamnionitis can be seen in Table 1.

Interestingly, pregnancies with chorioamnionitis resulting in the births of newborns <2,500 g have been noted to contain significantly higher frequencies of anaerobic organisms such as *Bacteroides bivius* and *Fusobacterium* than term controls but similar frequencies of gram-negative enterics and group B streptococci. One can speculate that these anaerobes may asymptomatically ascend to the uterine cavity and initiate preterm labor and delivery through deciduitis and expression of humoral factors such as tumor necrosis factor (TNF), interleukins, and other cytokines. Bejar et al.[17] have noted that phospholipase A2 activity may be highest in anaerobic isolates and that this resultant activity could result in the generation of prostaglandins from the decidua or chorion and lead to uterine contractions.

Even though amniotic-fluid cultures are positive for group B streptococci or *E. coli* in only 20% of patients with clinical chorioamnionitis, these 2 organisms account for two-thirds of maternal and neonatal bacteremia.[18] When either group B streptococci or *E. coli* is present in the amniotic fluid, 25–33% of the mothers or neonates will manifest bacteremia. Rarely will anaerobic bacteria be found in the maternal or neonatal bloodstream. It is unclear if the low incidence of anaerobic bacteremia reflects the true clinical state or greater difficulty in the laboratory in detecting these organisms.

In addition to the initiation of labor, chorioamnionitis may lead to bacteremia, adult respiratory distress syndrome (ARDS), disseminated intravascular coagulation (DIC), acute renal failure, or septic shock secondary to the production of endotoxin and various humoral factors and cytokines (Fig. 1). The elucidation of these humoral factors during the past 10 years has greatly increased our understanding of the pathogenesis of the sepsis syndrome. Arachidonic-acid metabolites such as the leukotrienes, prostaglandins, and thromboxanes have complex effects on cellular physiology. Thromboxane A2 has been shown to cause platelet and neutrophil aggregation and activation, vasoconstriction of vascular beds, and bronchoconstriction. Prostacyclin has been shown to inhibit platelet adhesiveness, stimulate the release of cytokines, inhibit gastric-acid secretion, and relax smooth muscle.[19] Increased levels of TNF and interleukin-1 are associated with increased mortality rates in septic shock. Initial investigations into the use of anticytokine antibodies in the management of sepsis have been encouraging. Randomized control trials have shown improvements in survival when septic patients received anti-TNF antibody, interleukin-6 receptor antagonist, and anti-endotoxin antibodies.[20] However, these data must be considered preliminary, and further research is still needed.

### HOST DEFENSES

It is unclear if amniotic fluid contains bacteriostatic or bacteriocidal substances. The simple empiric observation that clinical chorioamnionitis is an infrequent event in patients with intact membranes led early investigators to presume that amniotic fluid

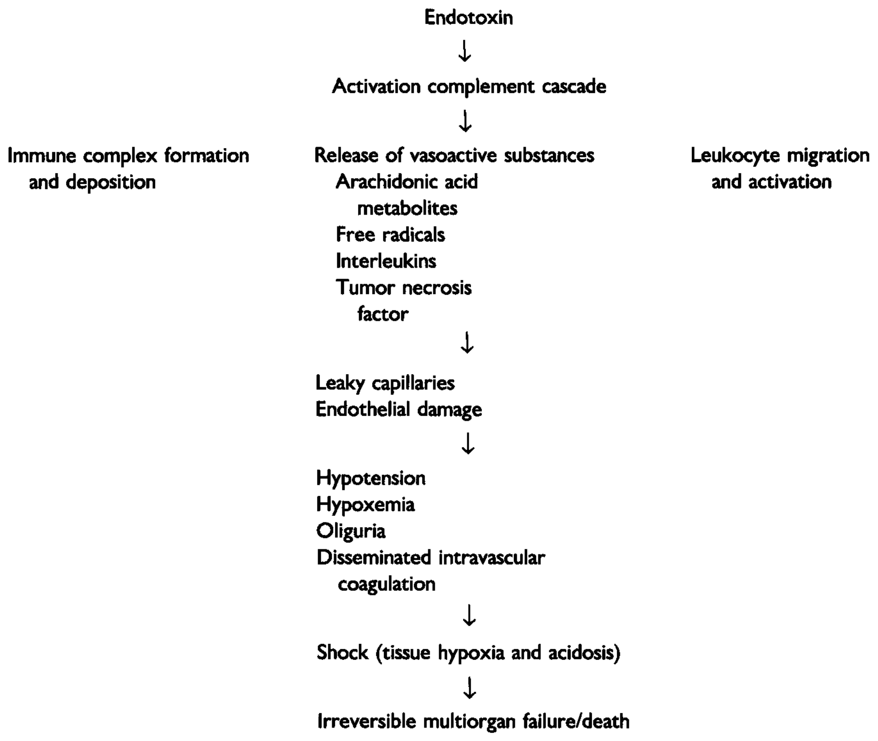

Fig. 1. Pathophysiology of multiorgan failure in chorioamnionitis.

contains antibacterial agents. In addition, studies have demonstrated that a strong correlation exists between the degree of oligohydramnios after preterm premature rupture of the membranes and clinical chorioamnionitis.[21] Investigators have attempted to document the presence of chemotactins, lysozymes, immunoglobulins, transferrin, and a zinc-peptide complex. None of these has conclusively or repeatedly been shown to modify clinical infection.[22]

### RISK FACTORS

The classically described risk factors for clinical chorioamnionitis are summarized in Table 2. In 1989, Soper et al.[6] and Newton et al.[5] prospectively evaluated risk factors by multiple regression analysis. Interestingly, several of the "established" risk factors noted in Table 2 did not meet the criteria for independent risk status. The rates of clinical chorioamnionitis were noted to be 4% and 10%, respectively, and these high rates may have contributed to the elimination of some factors classically associated with clinical chorioamnionitis. These investigators established probability curve paradigms that may serve as models for future research (Tables 3 and 4). Their observation that most patients developing clinical chorioamnionitis entered high-risk categories for several hours prior to the actual manifestation of chorioamnionitis is especially intriguing, as intervention during the prodromal period could theoretically modify the infectious process.

TABLE 2. Traditional risk factors for intraamniotic infection

| |
|---|
| Duration of rupture of membranes |
| Internal fetal monitoring |
| Number of vaginal examinations |
| Duration of labor |
| Young maternal age |
| Low gravidity and parity |
| Low socioeconomic status |
| Requirement of oxytocin augmentation |

### DIAGNOSIS

The diagnosis of clinical chorioamnionitis is suggested by the presence of fever in a gravid patient without evidence of urinary, respiratory, or other localized infection. Ruptured membranes are typically present but are not essential for the diagnosis to be made. Most authors have noted that the fre-

**TABLE 3. Probability of intraamniotic infection given varying combinations of risk factors[a]**

| No. of vaginal examinations | Duration of ruptured membranes (h) | Total labor (h) | Probability |
|---|---|---|---|
| 2 | 5.25 | 6.0 | 0.03 |
| 5 | 12.00 | 16.0 | 0.16 |
| 6 | 16.00 | 21.0 | 0.32 |
| 8 | 21.75 | 29.0 | 0.63 |
| 13 | 88.00 | 29.0 | 0.95 |

[a]Data from Soper et al.[6]

**TABLE 4. Criteria predicting a probability of intraamniotic infection of 20% or greater[a]**

| Internal monitoring (h) | | Membrane rupture (h) |
|---|---|---|
| 0 | with | ≥ 60 |
| 1 | with | ≥ 48 |
| 2 | with | ≥ 36 |
| 3 | with | ≥ 28 |
| 4 | with | ≥ 14 |
| 5 | with | ≥ 6 |
| 6 | with | ≥ 6 |

[a]Data from Newton et al.[5]

quency of associated physical findings is low. Hauth et al.[23] noted, in more than 100 cases of clinical chorioamnionitis, that fever was present in >99%, fetal tachycardia in 82%, maternal tachycardia in 19%, uterine tenderness in 16%, and malodorous amniotic fluid in 9%. The diagnosis is established clinically. Laboratory assessments such as leukocytosis with a left shift, increased C-reactive protein, leukocyte esterase assays, and gas chromatography are nonspecific, insensitive, or expensive and cumbersome, and therefore not clinically useful. Infrequently, the diagnosis may be suggested in an afebrile, laboring gravida by signs of fetal distress such as fetal tachycardia and decreased variability. Rarely, the diagnosis will be made in an afebrile, nonlaboring patient with intact membranes who presents with decreased fetal movement and an abnormal biophysical assessment.

### MANAGEMENT

The management of clinical chorioamnionitis must include an assessment of the clinical severity of the maternal and fetal infection, an awareness of the likelihood of vaginal delivery, an estimation of the anticipated timing of the delivery, and an anticipation of the postpartum and postnatal complications in chorioamnionitis (Fig. 2). Though the risk of maternal death due to chorioamnionitis in modern obstetrics is extremely low, the risk for significant morbidity is real. As previously discussed, endotoxemia may result in endothelial damage and lead to hypoxemia due to a capillary leak syndrome. Bacteremia may lead to septic shock with resultant ARDS, DIC, and acute renal failure. Close attention must be paid to the volume status and clinical signs of disease progression such as hypotension, tachycardia, tachypnea, and oliguria. The management of these complications must be undertaken with the appropriate critical-care consultation in an intensive care setting where hemodynamic observations and manipulations may be made. The interested reader is directed to other works regarding the management of these conditions, as a description of them is beyond the scope of this review.

The initial laboratory assessment should include blood and urine cultures, CBC and differential, and serum chemistries including BUN and creatinine. Maternal blood cultures are recommended, as up to 10% of patients will exhibit bacteremia.[24] Coagulation studies should be performed if thrombocytopenia or a bleeding diathesis is evident. Continuous fetal heart rate monitoring of all viable fetuses should be initiated at the time of diagnosis. Fetal tachycardia may be a sign of fetal sepsis or may simply reflect an appropriate cardiovascular response to endogenous maternal pyrogens such as the interleukins and TNF. The loss of variability typically seen at the fetal heart rate baselines of >180 beats/min will prevent the obstetrician from making an accurate assessment of fetal acid base status, and serial scalp samplings may be required to document that acidosis is not developing. It has been hypothesized that cord-blood gases indicating pre-acidosis or a mild metabolic acidosis may be a marker for neonatal bacteremia, but investigators testing this hypothesis have not been able to validate this theory.[25]

Infected fetuses can rapidly decompensate during labor. For example, at the time of the mother's presentation to the labor floor, the fetus may exhibit a fetal heart tracing with normal variability, baseline, and periodic changes. After several hours

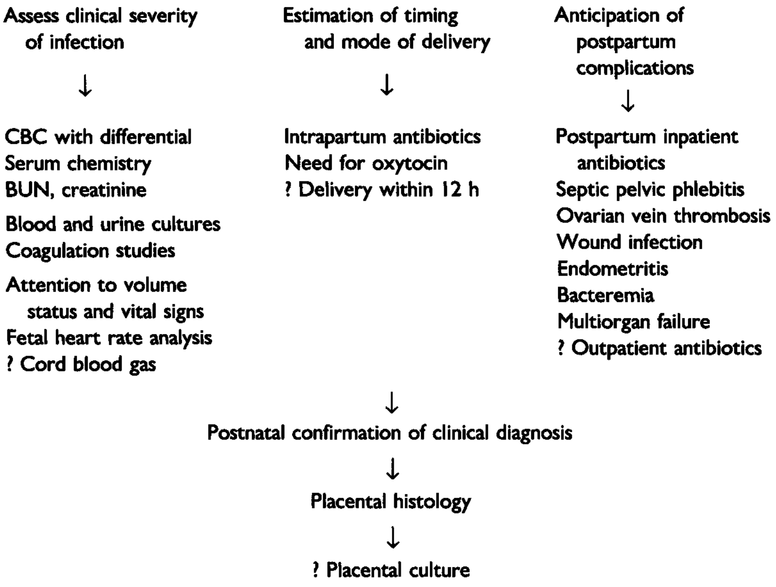

**Fig. 2. Evaluation and management of clinical chorioamnionitis.**

of labor, the fetus may manifest a new onset baseline tachycardia with decreased variability but no decelerations. Thirty minutes later, persistent late decelerations may be noted without a change in variability. Sixty minutes after the first tracing, there may be absent variability. At delivery or shortly thereafter, such a neonate may be septic, clinically depressed, and subsequently developmentally delayed.

Clinical chorioamnionitis has been associated with an increased risk for abnormal labor progression.[26] However, initial studies did not explain whether the chorioamnionitis causes or results from a protracted labor. Silver et al.[27] compared the labor progress in afebrile, laboring nulliparas at term with and without positive intraamniotic cultures obtained through serial intrauterine pressure catheter aspirations. Nulliparas with persistent high-virulent isolates (but no evidence of clinical chorioamnionitis) had significantly lower cervical dilation rates, despite a higher oxytocin infusion rate, and a higher cesarean delivery rate. They suggested that the presence of high-virulence bacteria in the amniotic fluid predisposes to hypocontractility and poor cervical dilation. In vitro evidence to support this suggestion has been reported by Leek et al.[28] They noted that a number of different bacterial isolates reduced or eradicated the spontaneous motility of myometrial strips in vitro. In addition, they noted that physiologic doses of oxytocin were unable to overcome this inhibitory effect.

Once a diagnosis of clinical chorioamnionitis has been established, delivery is indicated to remove the infected fetus and placenta from the uterus and reduce the risk of sepsis to the fetus and mother. However, the optimal time frame in which to deliver the fetus is unknown. Few data exist to describe the most appropriate interval from either diagnosis to delivery or antibiotic administration to delivery. Although clinicians in the 1970s recommended that delivery be effected within 1–2 h after the diagnosis, recent data have not supported this recommendation. Gibbs et al.[29] studied 171 gravidas with clinical chorioamnionitis and noted no differences in the percentages of pregnancies with maternal or neonatal bacteremia when the diagnosis-to-delivery intervals were arbitrarily broken into 4-h increments. However, $<10\%$ of their fetuses were delivered $>12$ h after the diagnosis. Hauth et al.[23] studied 103 gravidas with clinical chorioamnionitis in whom no differences in neonatal outcome were noted when the diagnosis-to-delivery intervals were increased from 1 h up to 10 h. Again, however, only 2% of their patients were delivered after 10 h. It appears from the above data that a diagnosis-to-delivery interval of up to 12 h is not associated with increased neonatal morbidity

(assuming that the mother is receiving parenteral antibiotics). However, the risks to the fetus, if a delay beyond 12 h occurs, are simply unknown. In the only prospective, randomized control trial to date regarding the therapeutic management of clinical chorioamnionitis, Gibbs et al.[30] excluded patients who were <4 cm dilated at the time of diagnosis for fear of a prolonged labor course and resultant adverse perinatal outcome.

Until the late 1980s, no consensus of opinion existed as to the most opportune time to initiate antibiotics after making the diagnosis of clinical chorioamnionitis. Prior to this time, many obstetricians chose to defer antibiotics until after the cord was clamped so as not to interfere with the diagnosis and management of the neonate. Based on 2 retrospective studies and 1 prospective study at large indigent institutions, popular opinion appears to have changed. Sperling et al.[31] published a retrospective examination of 257 term gravidas with clinical chorioamnionitis and noted that the incidence of neonatal bacteremia was significantly lower in newborns who received intrapartum antibiotics vs. those receiving antibiotics after cord clamping (2.8% vs. 20%, respectively). Gilstrap et al.[32] noted a similar trend but without statistical significance. In these retrospective studies, the patients who did not receive antibiotics until after delivery had significantly lower maximum temperatures and shorter diagnosis-to-delivery intervals. These facts serve to confirm the efficacy of intrapartum treatment, since the post-delivery treatment group should have had even lower neonatal sepsis rates. The antibiotics used in these studies typically included a penicillin or derivative (with or without an aminoglycoside) or a second-generation cephalosporin.

Gibbs et al.[30] have published the only randomized, controlled trial to date evaluating the usefulness of intrapartum antibiotics in the prevention of neonatal sepsis. The trial was stopped after only half of the planned patients were enrolled because an independent safety committee's interim analysis had noted statistically significant differences in the rates of neonatal sepsis and pneumonia between the treated and untreated groups (0% vs. 33%). No differences in Apgar scores were noted.

Although no data exist to prove or disprove the usefulness of anaerobic coverage during the intrapartum treatment of chorioamnionitis, the rarity of anaerobic neonatal sepsis has precluded the routine use of intrapartum clindamycin or an equivalent antibiotic. However, it is commonplace at our institution to add clindamycin during the intrapartum period if the mother shows clinical signs of deterioration or high fever (temperature >39%). The ideal antibiotic regimen for intrapartum usage is unknown at this time. Antibiotics cross the placenta in varying concentrations, but the measurement of antibiotic levels in various maternal and fetal compartments suggests that ampicillin and gentamycin have high cord blood/maternal blood ratios.[33] In our institution, we typically administer 2 g of ampicillin every 6 h and 100 mg of gentamycin every 8 h (after a 120-mg loading dose) to gravidas with suspected chorioamnionitis. We administer 900 mg of clindamycin every 8 h instead of ampicillin if the patient reports a penicillin allergy. Although ampicillin and gentamycin are considered the "gold standard," newer broad-spectrum agents such as ticarcillin or ampicillin-sulbactam demonstrate similar bacterial coverage. Nevertheless, large series have not yet been reported to ensure their clinical equivalence.

Studies have repeatedly demonstrated that cesarean delivery increases the maternal morbidity in clinical chorioamnionitis. Koh et al.[34] reported a 43% incidence of endometritis after cesarean delivery vs. only an 11% incidence after vaginal delivery. Gogoi[35] reported that 13 of 14 maternal deaths as a result of chorioamnionitis occurred after cesarean delivery. Gravidas with clinical chorioamnionitis who are delivered abdominally are at increased risk for postoperative peritonitis, postpartum endometritis, septic pelvic thrombophlebitis, ovarian vein thrombosis, and wound infection. Yonekura et al.[26] reported no difference in postpartum morbidity when comparing transperitoneal vs. extraperitoneal cesarean delivery. IV antibiotics should be continued after cesarean delivery until fever has been absent for 24–48 h and the clinical signs and symptoms are resolving. Additional anaerobic coverage such as that obtained with clindamycin or metronidazole should be added after cord clamping if not previously administered during the intrapartum period. This additional coverage is given to ensure adequate coverage of the anaerobic organisms traditionally associated with postpartum endometritis. The lower incidence of postpartum endometritis after a vaginal delivery makes the need for the continuation of IV antibiotics after vaginal delivery less certain.

Recent studies have demonstrated that there may be no need for a course of oral antibiotics after a patient is discharged from the hospital in the case of an uncomplicated chorioamnionitis or endometritis.[37,38] Gravidas presenting with bacteremia, immunosuppression, HIV positivity, concurrent wound or other infection, abscess, or septic pelvic phlebitis have been excluded from these studies. These patients deserve full courses of the traditionally described antibiotic regimens.

The treatment of clinical chorioamnionitis in the gravida with ruptured membranes using antibiotics alone and without an effort to deliver her poses risks to both mother and fetus. However, the successful eradication of clinical chorioamnionitis without delivery in gravidas with intact membranes and listeriosis has been reported in all 3 trimesters.[39–41] In the absence of symptoms of an upper respiratory infection, we believe that the gravida presenting with fever, a flu-like illness, and uterine contractions can be maintained on IV ampicillin until blood-culture negativity has been confirmed and listeriosis ruled out. Pregnant patients should avoid the ingestion of raw meats, dairy products such as soft cheeses (brie or feta), and ready-to-eat packaged meats such as patés and sandwich meats.[42]

### NEONATAL MANAGEMENT

Chorioamnionitis presents a number of threats to the fetus and neonate. Sepsis rates of 1–6% are seen, and congenital pneumonia may be found in as many as 25% of neonates.[24,34] The mortality rate of neonatal septicemia has been described as 25–50%, although more recent series have noted lower rates. Morales et al.[43] reported that mental and physical development scores were not different in preterm gestations complicated by chorioamnionitis after controlling for ARDS, intraventricular hemorrhage, and birth weight. However, because premature newborns delivered through a clinically infected uterus exhibit higher rates of ARDS and intraventricular hemorrhage vs. gestational age- and weight-matched infants, their physical and developmental scores and mortality rates will be worse than their noninfected counterparts.[44]

Although intrapartum antibiotics have been touted as a method of reducing the risk of neonatal sepsis, they by no means reduce this risk to zero. In a retrospective series of nearly 100 newborns positive for early-onset group B streptococcal bacteremia, 19% of these babies had received intrapartum antibiotics at a median age of 4 h prior to delivery.[45] Seventy-two percent of these newborns were born to mothers suspected of having chorioamnionitis. In addition, both the large data set from Gilstrap et al.[32] and a small series from McDuffie et al.[46] suggest that intrapartum ampicillin may result in neonatal sepsis due not to group B streptococcus, but rather to more virulent and resistant organisms. Intrapartum chemoprophylaxis has never been to shown to prevent late-onset group B streptococcal infection.

The management of a term neonate delivered through an infected uterus is controversial. Wiswell et al.,[47] in a survey of 75 teaching institutions, noted that only 30% of the respondents initiate therapy of the asymptomatic term neonate and only 70% perform a laboratory or x-ray evaluation. At our institution, we evaluate the asymptomatic term neonate delivered through a clinically infected uterus with a CBC and blood culture immediately after birth. Antibiotics are not begun if the mother received intrapartum antibiotics unless the neonate manifests signs of sepsis or the blood culture returns positive. Asymptomatic preterm newborns are treated with a 7–10 day course of IV ampicillin and gentamycin for presumptive sepsis. Symptomatic newborns (whether term or preterm) receive a 10–21 day course depending on the infant's clinical course and culture results.

### SUMMARY

Clinical chorioamnionitis continues to be associated with significant neonatal and maternal morbidity and mortality. Research during the last 20 years has enabled us to characterize the epidemiology and molecular pathophysiology leading to this infection. Unfortunately, we currently cannot establish the diagnosis until the infection is clinically evident, resulting in alterations in the normal maternal-fetal homeostasis. Current topics of research include assessments of primary prevention such as vaccination with group B streptococcal antigen, early identification of at-risk parturients using clinical paradigms or rapid detection antigen assays, and determination of the optimal type and mode of delivery of antibiotics.

### REFERENCES

1. Mueller-Heubach E, Rubinstein DN, Shen-Schwarz S: Histologic chorioamnionitis and preterm delivery in different patient populations. Obstet Gynecol 75:662–626, 1990.
2. Russell KP, Anderson GV: Aggressive management of ruptured membranes. Am J Obstet Gynecol 83:930, 1962.
3. Gibbs CE, Locke WE: Maternal deaths in Texas 1969–1973: A report of 501 consecutive deaths from Texas Medical Association Committee on Maternal Health. Am J Obstet Gynecol 78:373, 1976.
4. Mertz KJ, Parker AL, Halpin GJ: Pregnancy-related mortality in New Jersey, 1975 to 1989. Am J Public Health 82:1085–1088, 1992.
5. Newton ER, Prihoda TJ, Gibbs RS: Logistic regression analysis of risk factors for intra-amniotic infection. Obstet Gynecol 73:571–575, 1989.
6. Soper DE, Mayhall G, Dalton HP: Risk factors for intra-amniotic infection: A prospective epidemiologic study. Am J Obstet Gynecol 161:562–568, 1989.
7. Rhoads GG, Jackson LG, Schlesselman E, et al: The safety and efficacy of chorionic villus sampling for early prenatal diagnosis of cytogenetic abnormalities. N Engl J Med 320:609–617, 1989.
8. Wilkins I, Mezrow G, Lynch L, Bottone EJ, Berkowitz RL: Amnionitis and life-threatening respiratory distress after percutaneous umbilical blood sampling. Am J Obstet Gynecol 160:427–428, 1989.
9. Golan A, Barnan R, Wexler S, et al: Incompetence of the uterine cervix. Obstet Gynecol Surv 44:96–107, 1989.
10. Harger JH. Comparison of success and morbidity in cervical cerclage procedures. Obstet Gynecol 56:543–548, 1980.
11. Schuchat A, Deaver KA, Wenger JD, et al: Role of foods in sporadic listeriosis: Case-control study of dietary risk factors. JAMA 267:2041–2045, 1992.
12. Monif GRG: Antenatal group A streptococcal infection. Am J Obstet Gynecol 123:213–214, 1975.
13. Duff P, Gibbs RS: Acute intra-amniotic infection due to *Streptococcus pneumoniae*. Obstet Gynecol 61:25s–27s, 1983.
14. Smith LG, Summers PR, Miles RW, Biswas MJ, Pernoll ML: Gonococcal chorioamnionitis associated with sepsis: A case report. Am J Obstet Gynecol 160:573–574, 1989.
15. Gibbs RS, Blanco JD, St Clair PJ, Castaneda YS: Quantitative bacteriology of amniotic fluid from patients with clinical intra-amniotic infection at term. J Infect Dis 145: 1–8, 1982.
16. Gravett MG, Eschenbach DA, Speigel-Brown CA, Holmes KK: Rapid diagnosis of amniotic fluid infection by gas-liquid chromatography. N Engl J Med 306:725–728, 1982.
17. Bejar R, Curbelo V, Davis C, et al: Premature labor. I. Bacterial sources of phospholipids. Obstet Gynecol 57: 479, 1981.
18. Gibbs RS, Duff P: Progress in pathogenesis and management of clinical intra-amniotic infection. Am J Obstet Gynecol 164:1317–1326, 1991.
19. Pinsky MR, Matuschak GM: Multi systems organ failure: Failure of host defense homeostasis. Crit Care Clin 5:199–221, 1989.
20. Rackow EC, Astiz ME: Mechanisms and management of septic shock. Crit Care Clin 3:210–237, 1993.
21. Vintzileos A, Campbell W, Nochimson D, Connolly M, Fuenfer M, Hoehn G: The fetal biophysical profile in patients with premature rupture of membranes: An early predictor of fetal infection. Am J Obstet Gynecol 152: 510, 1985.
22. Ford LC, DeLange RJ, Lebherz TB: Identification of a bactericidal factor (B-lysin) in amniotic fluid at 14 and 40 weeks' gestation. Am J Obstet Gynecol 127:788–798, 1977.
23. Hauth JC, Gilstrap LC, Hankins GD, Connor KD: Term maternal and neonatal complications of acute chorioamnionitis. Obstet Gynecol 66:59–62, 1985.
24. Yoder RP, Gibbs RS, Blanco JD, et al: A prospective controlled study of maternal and perinatal outcome after intra-amniotic infection at term. Am J Obstet Gynecol 145:695–698, 1983.
25. Ramin S, Maberry M, Gilstrap LC, et al: Fetal acidemia in pregnancies with chorioamnionitis. Obstet Gynecol 76: 351–354, 1990.
26. Duff P, Sanders R, Gibbs RS: The course of labor in term patients with chorioamnionitis. Am J Obstet Gynecol 147:391, 1983.
27. Silver RK, Gibbs RS, Castillo M: Effect of amniotic fluid bacteria in the course of labor in nulliparous women at term. Obstet Gynecol 68:587–592, 1986.
28. Leek BF, McDonald D, Vaughan J: The spontaneous motility of human myometrial strips in vitro and its modification by some bacterial extracts. J Physiol 324:42p, 1981.
29. Gibbs RS, Castillo MS, Rodgers PJ: Management of acute chorioamnionitis. Am J Obstet Gynecol 136:709–713, 1980.
30. Gibbs RS, Dinsmoor NJ, Newton ER, Ramamurthy RS: A randomized trial of intrapartum versus immediate postpartum treatment of women with intra-amniotic infection. Obstet Gynecol 72:823–827, 1988.
31. Sperling RS, Ramamurthy RS, Gibbs RS: A comparison of intrapartum versus immediate postpartum treatment of intra-amniotic infection. Obstet Gynecol 70:861–865, 1987.
32. Gilstrap LC, Leveno KJ, Cox SM, Burris JS, Mashburn M, Rosenfeld CR: Intrapartum treatment of acute chorioamnionitis: Impact on neonatal sepsis. Am J Obstet Gynecol 159:579–583, 1988.
33. Gilstrap LC, Bawdon RE, Burris J: Antibiotic concentration in maternal blood, cord blood, and placental membranes in chorioamnionitis. Obstet Gynecol 72:124–125, 1988.
34. Koh KS, Chan FH, Monfared AH, Ledger WJ, Paul RH: The changing perinatal and maternal outcome in chorioamnionitis. Obstet Gynecol 53:730–734, 1979.
35. Gogoi MP: Maternal mortality from cesarean section in infected cases. Br J Obstet Gynaecol 78:373, 1971.
36. Yonekura ML, Wallace R, Eglinton G: Amnionitis—optimal operative management: Extraperitoneal cesarean section vs low cervical transperitoneal cesarean section (abstract). In: Proceedings of the Third Annual Meeting of the Society of Perinatal Obstetricians, San Antonio, Texas, January 1983. San Antonio: Society of Perinatal Obstetricians, 1983.

37. Stovall TG, Amborse SE, Ling FW, Anderson GD: Short course antibiotic therapy for the treatment of chooioamnionitis and postpartum endomyometritis. Am J Obstet Gynecol 159:404–407, 1988.
38. Morales WJ, Collins EM, Angel JC, Knuppel RA: Short course of antibiotic therapy in treatment of postpartum endomyometritis. Am J Obstet Gynecol 161:568–572, 1989.
39. Cruikshank DP, Warenski JC: First trimester maternal *Listeria monocytogenes* sepsis and chorioamnionitis with normal neonatal outcome. Obstet Gynecol 73:469–471, 1989.
40. Hume OS: Maternal *Listeria monocytogenes* septicemia with sparing of the fetus. Obstet Gynecol 27:390–396, 1961.
41. Lennon D, Lewis B, Mantell C, et al: Epidemic perinatal listeriosis. Pediatr Infect Dis J 3:30–34, 1984.
42. Yonekura ML, Mead PB: Listeria infections. Contemp OB/GYN 83–86, 1992.
43. Morales WJ: The effect of chorioamnionitis on the developmental outcome of preterm infants at one year. Obstet Gynecol 70:183–186, 1987.
44. Hardt NS, Kostenbauder M, Ogburn M, et al: Influence of chorioamnionitis on long-term prognosis in low birth weight infants. Obstet Gynecol 65:5–10, 1985.
45. Ascher DP, Becker JA, Yoder BA, et al: Failure of intrapartum antibiotics to prevent culture-proved neonatal group B streptococcal sepsis. J Perinatol 13:212–216, 1993.
46. McDuffie RS, McGregor JA, Gibbs RS: Adverse perinatal outcome and resistant enterobacteriaceae after antibiotic usage for premature rupture of the membranes and group B streptococcus carriage. Obstet Gynecol 82:487–489, 1993.
47. Wiswell TE, Stoll BJ, Tuggle JM: Management of asymptomatic, term gestation neonates born to mothers treated with intrapartum antibiotics. Pediatr Infect Dis J 9:826–831, 1990.
